# Descending serotonergic facilitation and the antinociceptive effects of pregabalin in a rat model of osteoarthritic pain

**DOI:** 10.1186/1744-8069-5-45

**Published:** 2009-08-07

**Authors:** Wahida Rahman, Claudia S Bauer, Kirsty Bannister, Jean-Laurent Vonsy, Annette C Dolphin, Anthony H Dickenson

**Affiliations:** 1 Department of Neuroscience, Pharmacology and Physiology, University College London, Gower Street, London, WC1E 6BT, UK

## Abstract

**Background:**

Descending facilitation, from the brainstem, promotes spinal neuronal hyperexcitability and behavioural hypersensitivity in many chronic pain states. We have previously demonstrated enhanced descending facilitation onto dorsal horn neurones in a neuropathic pain model, and shown this to enable the analgesic effectiveness of gabapentin. Here we have tested if this hypothesis applies to other pain states by using a combination of approaches in a rat model of osteoarthritis (OA) to ascertain if 1) a role for descending 5HT mediated facilitation exists, and 2) if pregabalin (a newer analogue of gabapentin) is an effective antinociceptive agent in this model. Further, quantitative-PCR experiments were undertaken to analyse the α_2_δ-1 and 5-HT3A subunit mRNA levels in L3–6 DRG in order to assess whether changes in these molecular substrates have a bearing on the pharmacological effects of ondansetron and pregabalin in OA.

**Results:**

Osteoarthritis was induced via intra-articular injection of monosodium iodoacetate (MIA) into the knee joint. Control animals were injected with 0.9% saline. Two weeks later *in vivo *electrophysiology was performed, comparing the effects of spinal ondansetron (10–100 μg/50 μl) or systemic pregabalin (0.3 – 10 mg/kg) on evoked responses of dorsal horn neurones to electrical, mechanical and thermal stimuli in MIA or control rats. In MIA rats, ondansetron significantly inhibited the evoked responses to both innocuous and noxious natural evoked neuronal responses, whereas only inhibition of noxious evoked responses was seen in controls. Pregabalin significantly inhibited neuronal responses in the MIA rats only; this effect was blocked by a pre-administration of spinal ondansetron. Analysis of α_2_δ-1 and 5-HT3A subunit mRNA levels in L3–6 DRG revealed a significant increase in α_2_δ-1 levels in ipsilateral L3&4 DRG in MIA rats. 5-HT3A subunit mRNA levels were unchanged.

**Conclusion:**

These data suggest descending serotonergic facilitation plays a role in mediating the brush and innocuous mechanical punctate evoked neuronal responses in MIA rats, suggesting an adaptive change in the excitatory serotonergic drive modulating low threshold evoked neuronal responses in MIA-induced OA pain. This alteration in excitatory serotonergic drive, alongside an increase in α_2_δ-1 mRNA levels, may underlie pregabalin's state dependent effects in this model of chronic pain.

## Background

Osteoarthritis (OA), the most common form of arthritis [1,2], is a degenerative joint disease, usually of weight bearing joints (knees and hips), characterized by damage to the articular cartilage and subchondral bone, synovitis and capsular thickening. OA can develop following the loss of joint stability, hence its aetiology is diverse and multifactorial, and is associated with chronic debilitating joint pain, that can range from mild (dull aches) to severe (sharp stabbing pain). While pain is usually the main symptom and the first reason of complaint of OA patients, it is only recently that research into the mechanisms of OA pain has developed.

Current therapies do not include disease modifying drugs, thus analgesics remain as the first line treatment for OA. Starting with paracetomol, treatment is then followed by NSAIDS, opioids and steroids in line with disease progression and increasing pain severity. However, the therapeutic window and the level of pain relief with these therapies is often inadequate in a large proportion of patients. Thus it is important to identify the molecular mechanisms that induce and maintain the pain state in order to develop more effective therapeutic agents.

In OA patients, approximately 60–80% achieve pain relief after local anaesthetic treatment or surgical replacement of the affected joint, indicating peripheral mechanisms driving the pain [3-6], although in some patients central mechanisms are also thought to play a role and dysfunction of diffuse noxious inhibitory controls have been described [7]. These central mechanisms may overlap with those in other pain states such as neuropathy.

Alongside alterations in descending inhibitory controls, descending facilitation from the brainstem has been indicated to be a key mechanism underlying some forms of chronic pain [8-18]. For instance, we have previously demonstrated an enhanced descending facilitation, mediated by brainstem serotonergic pathways acting at pronociceptive spinal 5HT3 receptors, that contributes to the pathophysiological changes following nerve injury and cancer induced bone pain [13,19]. Therefore, in the present study the role of descending facilitation in a rat model of OA was assessed.

We have used a chemical model of OA pain, using monosodium iodoacetate (MIA), a glycolysis inhibitor, which has proved a reliable and consistent model of osteoarthritis that mimics pain in OA patients [20-23]. Following a single intra-articular MIA injection into the rat knee joint, a progressive degeneration of cartilage is seen [24] and these changes have been shown to be similar to the human disease [25,26]. Whilst painful symptoms are mostly associated with the area surrounding the affected joint, referred pain and tenderness can also occur in patients [27,28] and is apparent in the MIA rat model as hypersensitive responses to stimulation of the ipsilateral hindpaw [23].

Following a two week OA induction period we assessed the contribution of descending facilitation, using in vivo electrophysiological methods to compare the effects of spinal application of the selective 5HT3 receptor antagonist, ondansetron, on evoked dorsal horn neuronal responses in MIA and control rats. Additionally, we have previously shown that descending facilitation from the brainstem enables the anti-hyperalgesic actions of gabapentin following peripheral nerve injury [29]. This led us to assess the effects of pregabalin (a newer analogue of gabapentin) on spinal neuronal activity in MIA rats, to test whether descending serotonergic facilitation is an important mechanism enabling the analgesic effectiveness of pregabalin in this model of chronic pain. In these studies we have concentrated on secondary hyperalgesia in that we have examined responses evoked from the paw after knee MIA. Gabapentin has state-dependent actions, modulating abnormal activity and has been shown to be effective in human imaging studies in the presence of central sensitization [30].

## Results

Following intra-articular injection of MIA or saline, both groups of rats maintained good health, exhibiting normal weight gain and general level of activity with no signs of distress, and the final weight at time of electrophysiology was not different between the MIA (271 ± 7 g) or sham (275 ± 11 g) groups.

### Behavioural responses

**Figure 1:**
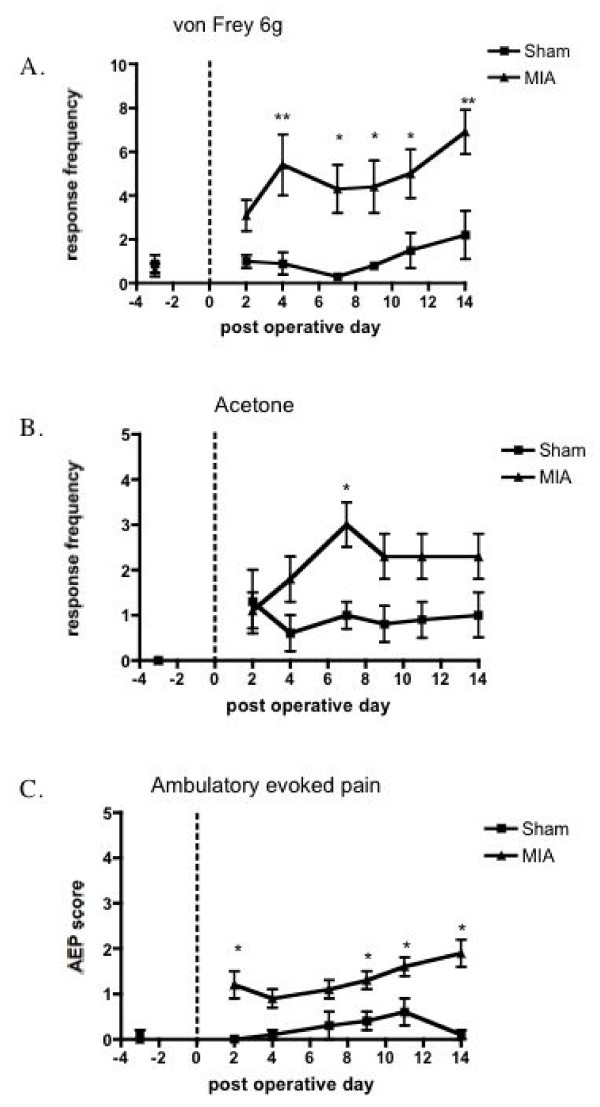
**Behavioural assessment from 3 days before until 14 days after MIA (n = 17) or saline injection (n = 16)**. A. mechanical and B. cooling stimuli applied to the ipsilateral hind paw. C. ambulatory evoked pain score. MIA injection into the knee resulted in behavioural hypersensitivity as evidenced by the significantly greater number of paw withdrawal responses to mechanical punctate and acetone application, and the observed pain scored on ambulation compared with the saline control rats. *p < 0.05, ** p < 0.01 compared with shams.

Probing the ipsilateral hind paw with von Frey hairs and application of acetone revealed behavioural signs of mechanical and cooling hypersensitivity, seen as a significant increase in ipsilateral hind paw withdrawal frequency to stimulation with von Frey filaments 1& 6 g and acetone application in the MIA group compared with the same responses in the sham-operated group (Fig. 1). Stimuli applied to the contra-lateral hind paw of MIA or sham rats produced little or no evoked hind paw withdrawal responses in either group (data not shown). Similarly, the ambulatory evoked pain score in the MIA rats was higher compared with the vehicle treated sham rats (Fig. 1). This behavioural hypersensitivity observed on the hind paw after MIA injection into the knee is indicative of secondary hyperalgesia and is likely due to central sensitization.

### Spinal cord electrophysiology – cell characterization

The evoked responses of ipsilateral deep dorsal horn neurones were characterised in MIA (n = 30) and saline injected sham (n = 34) rats (Table 1). The mean neuronal cell depth was not significantly different between experimental groups and corresponded to lamina V-VI of the spinal dorsal horn, thus enabling direct comparison of evoked neuronal responses. All neurones recorded in this study had receptive fields which included at least one toe on the hind paw and were classified as wide dynamic range, since they all responded to both light touch and noxious inputs (pinch and noxious heat), and responded to natural stimuli in a graded manner with increasing intensity.

**Table 1:** A comparison of the baseline electrophysiological responses of deep dorsal horn neurons in saline (n = 30) and MIA (n = 34) injected rats.

		**Sham (n = 30)**	**MIA (n = 34)**
	**Depth (μm)**	787 ± 50	730 ± 37

**Electrically-evoked Responses**	**C-fibre threshold (mA)**	0.76 ± 0.08	0.71 ± 0.07
	
	**Aβ-fibre evoked response (AP)**	157 ± 14	175 ± 13
	
	**Aδ-fibre evoked response (AP)**	136 ± 13	137 ± 12
	
	**C-fibre evoked response (AP)**	401 ± 31	475 ± 38
	
	**Post discharge (AP)**	257 ± 37	265 ± 34

	**Input (AP)**	334 ± 52	389 ± 37

	**Wind-up (AP)**	442 ± 58	452 ± 60

The electrical evoked responses are expressed as the mean number of action potentials (AP) ± S.E.M. evoked by a train of 16 electrical stimuli given at three times the threshold for C-fibre evoked neuronal response.

**Figure 2:**
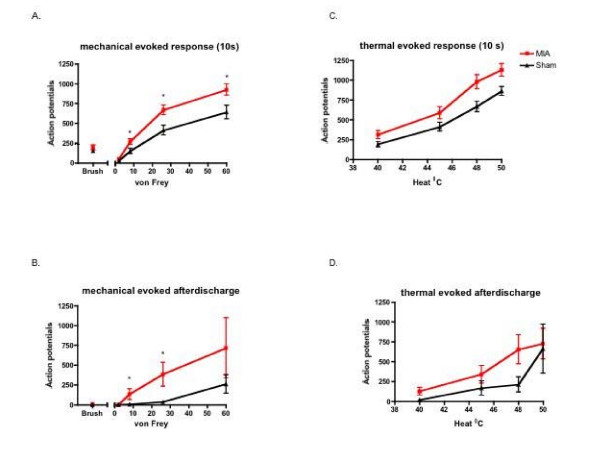
**Electrophysiological recordings of deep dorsal horn wide dynamic range neuronal responses in MIA (n = 30) and sham rats (n = 34)**. A) Mean evoked neuronal response of peripheral mechanical stimulation during the 10 second application period. B) Mean after-discharge response of neurons to mechanical stimulation. C) Mean evoked neuronal response of peripheral thermal stimulation during the 10 second application period D. Mean after-discharge response of neurons to thermal stimulation. MIA injection resulted in an overall increase in neuronal excitability. * p < 0.05 compared with sham control data.

The evoked neuronal responses to a train of electrical stimulation were not significantly different between groups (Table 1). In contrast, MIA injected rats exhibited an overall enhanced neuronal response, evoked during the 10-second application period, to mechanical stimulation in a stimulus intensity dependent manner; significant effects were seen in response to application of von Frey filaments 8–60 g (p < 0.05) (Fig. 2A). Furthermore, the mechanical evoked neuronal after-discharge, quantified as the total number of action potentials the neurone continued to fire after the removal of the stimulus period was greater in the MIA group (P < 0.03) (Fig. 2B). Enhanced thermal evoked neuronal responses were also seen in the MIA group compared with controls, although this did not quite reach significance (Fig. 2C, D). Thus, these results indicate a general hyperexcitable nature of spinal wide dynamic range neurones in segments adjacent to the primary insult in this model of OA (Fig. 2) indicative of central sensitization.

### Effects of spinal ondansetron administration on the evoked responses of dorsal horn neurones

**Figure 3:**
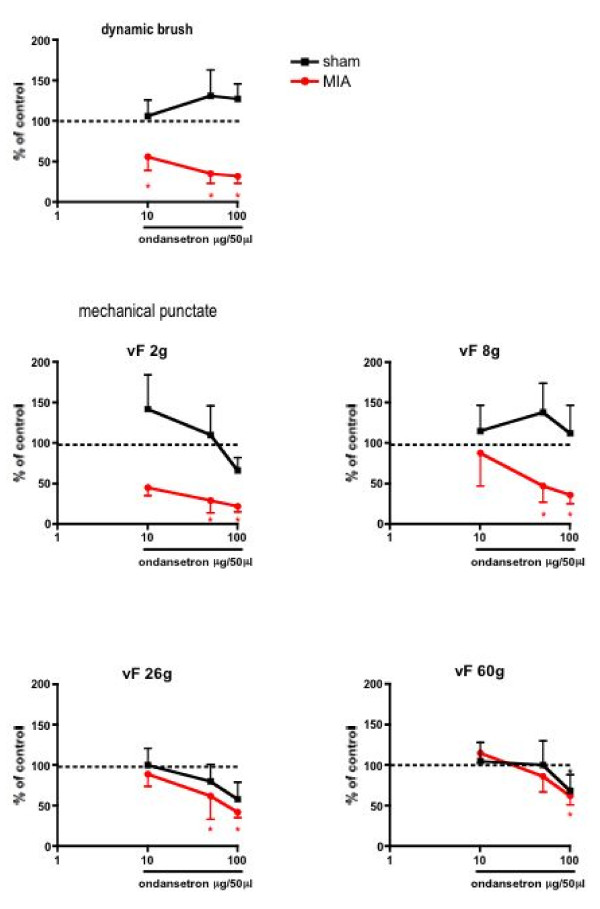
**Comparison of the effects of spinal administration of ondansetron on the evoked neuronal responses to dynamic brush and mechanical punctate stimulation in sham (n = 8) and MIA (n = 8) rats**. The neuronal responses evoked by dynamic brush, von Frey 2 and 8 g stimulation were significantly reduced by 50 and 100 μg of ondansetron in the MIA group. Ondansetron produced no significant effects on the mechanical evoked neuronal responses in the sham group. *p < 0.05 compared with pre- drug baseline control data.

**Figure 4:**
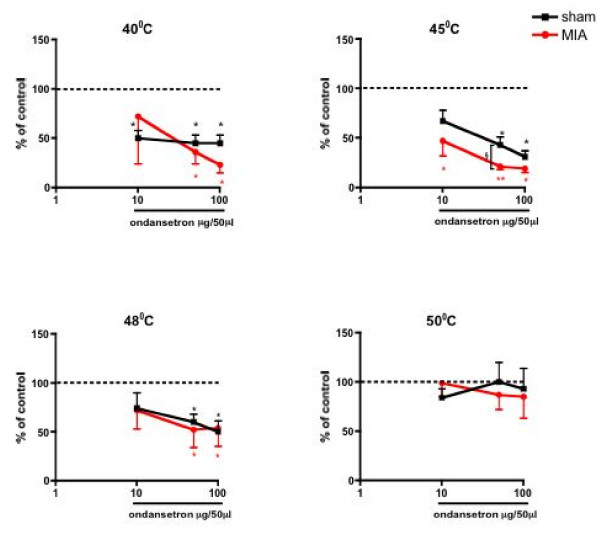
**Effects of spinal administration of ondansetron on the evoked neuronal responses to thermal stimulation of the peripheral receptive field area in sham (n = 8) and MIA (n = 8) rats**. Ondansetron at 5 μg produced a greater inhibition of the evoked response to 45°C in the MIA group compared with the control group; the effects of the drug on the other thermal stimuli tested were not different between groups. Data are expressed as the mean percentage of pre-drug control values ± S.E.M. *p < 0.05 **p < 0.01 compared with pre- drug baseline control data. §p < 0.05 compared with sham control group.

Spinal administration of ondansetron did not produce any significant effects on any of the electrically evoked neuronal responses in either the MIA or vehicle treated rats (data not shown). However, the same doses of spinal ondansetron administration resulted in a significant inhibition of some of the natural evoked responses in both groups (Figs. 3 &4).

In MIA injected rats, spinal ondansetron resulted in a dose-related inhibition of a range of natural evoked responses. The evoked neuronal response to stimulation of the peripheral receptive field with dynamic brush was significantly reduced at 10–100 μg (P < 0.05), and with von Frey 2 and 8 g were significantly reduced at 50 and 100 μg (P < 0.05) in the MIA rats compared with pre-drug control values. Strikingly, in contrast, ondansetron did not produce any significant inhibition of these low threshold evoked responses in the control group (Fig. 3). With regard to the evoked responses to noxious stimuli similar dose-related inhibitions were seen in MIA and vehicle-treated groups for both mechanical (von Frey 26 and 60 g) (Fig. 3), and thermal (45 and 48°C) modalities (Fig. 4). However, the inhibitory effect of ondansetron at 50 μg on the evoked response to 45°C heat was significantly greater in the MIA group when compared with the effect of the drug in the vehicle treated group (P < 0.05). Ondansetron at 10 μg produced a significant inhibition of the evoked neuronal response to 40°C in the sham control group, an effect not seen in the MIA treated group at this dose. The evoked responses to suprathreshold 50°C stimulation were not significantly altered by ondansetron in either group (Fig. 4).

These findings suggest that OA induces a novel contribution of 5HT3 receptor-mediated excitation of low threshold mechanical (dynamic brush and innocuous mechanical punctate) evoked neuronal responses and an enhancement of noxious thermal evoked responses to 45°C in MIA treated rats compared with control animals, since the antagonist was either without effect on these neuronal measures (low threshold mechanical) or had a lesser effect on noxious heat evoked responses (45°C) in the control group.

### Effects of systemic pregabalin administration on the evoked responses of spinal dorsal horn neurones

Since gabapentinoids have been shown to exert analgesic effects in a variety of models of hyperalgesia and allodynia [31,32], not simply neuropathy, and because we have previously demonstrated a link between 5HT3 receptor activity and efficacy of gabapentin [29], this led us to investigate the consequences of pregabalin administration on neuronal responses in the MIA model.

**Figure 5:**
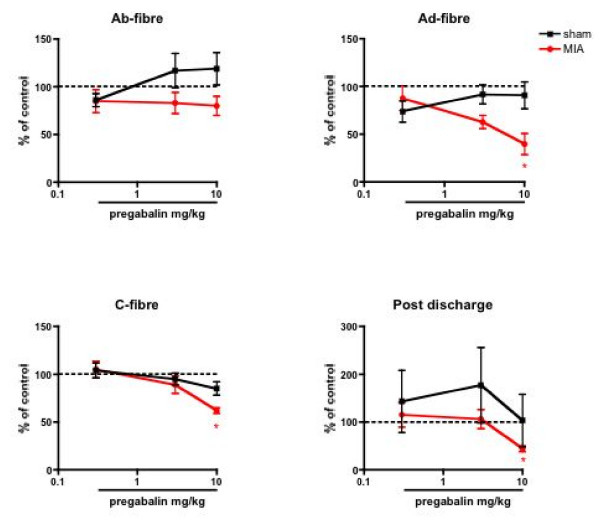
**A comparison of the effects of systemic adminsitration of pregabalin on the electrical evoked responses of spinal dorsal horn neurones in sham (n = 8) and MIA (n = 9) rats**. Pregabalin significantly attenuated the evoked A-delta, C-fibre and post discharge neuronal responses in the MIA group only. Data are expressed as the mean percentage of pre-drug control values ± S.E.M. * p < 0.05 significant difference compared with pre- drug baseline control data.

Systemic administration of pregabalin produced a significant inhibition of noxious electrical evoked responses in the MIA-treated group only. The A-delta fibre evoked response was clearly inhibited at 3 and 10 mg/kg pregabalin, with a maximum inhibition of 60 ± 11% of the pre-drug control response with the top dose. The C-fibre and post discharge evoked neuronal responses were also inhibited by the drug but to a much lesser extent, with a significant reduction compared to pre-drug control responses seen only at 10 mg/kg (Fig. 5).

**Figure 6:**
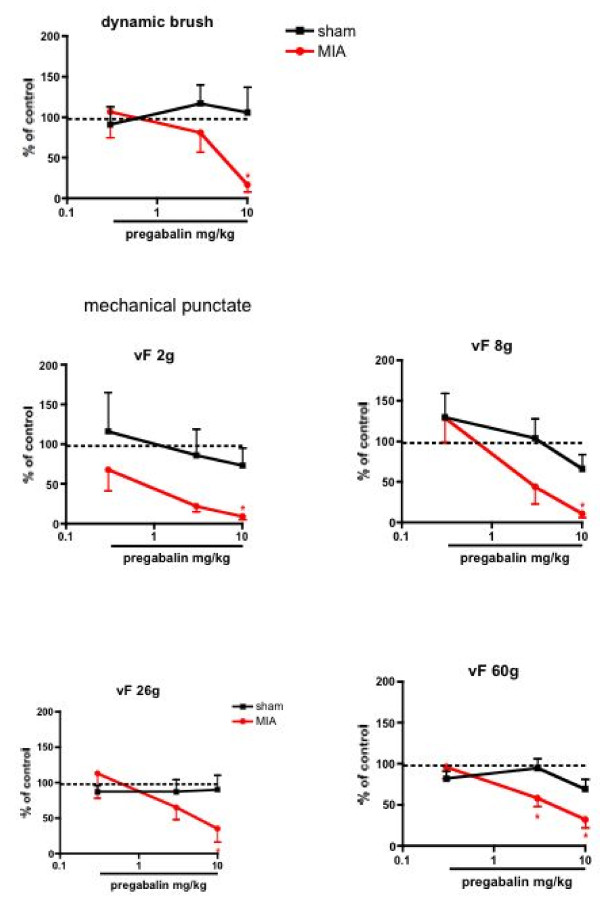
**Effects of systemic injection of pregabalin on the evoked neuronal responses to mechanical stimulation of the peripheral receptive field**. Pregabalin produced significant inhibition of dynamic brush and mechanical punctate evoked neuronal responses in the MIA treated group (n = 9) compared with pre-drug baseline responses. The drug produced little or no effect in the sham control group (n = 8). Data are expressed as the mean percentage of pre-drug control values ± S.E.M. * p < 0.05 significant difference compared with pre- drug baseline control data.

**Figure 7:**
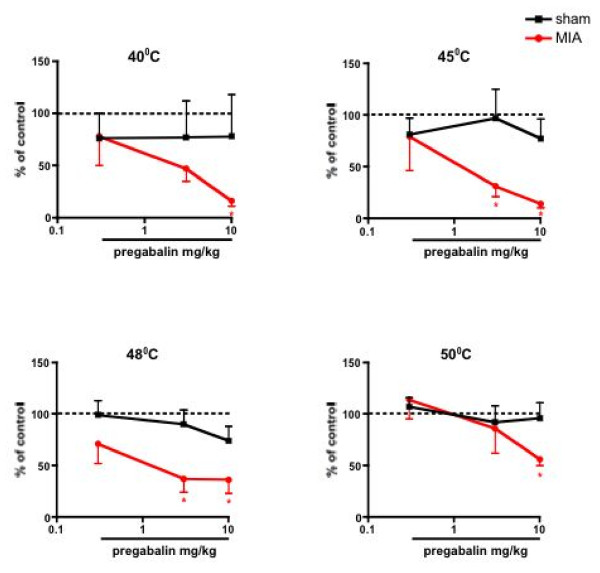
**Effects of systemic injection of pregabalin on the evoked neuronal responses to thermal stimulation of the peripheral receptive field**. Pregabalin significantly inhibited the thermal evoked neuronal responses in the MIA group (n = 9) compared with pre-drug baseline responses. The drug produced little or no effect in the sham control group (n = 8). Data are expressed as the mean percentage of pre-drug control values ± S.E.M. * p < 0.05 significant difference compared with pre- drug baseline control data.

The mechanical and thermal evoked responses in the MIA rats were inhibited similarly in a dose-related manner (P < 0.05, 1-way ANOVA) (Fig. 6 &7). In comparison, pregabalin produced no significant effects on any of the natural stimuli evoked neuronal measures in the sham controls when compared with pre-drug control neuronal responses, (Fig. 5, 6, 7) illustrating state-dependency of the drug

### Antagonism of spinal 5-HT3 receptors results in the loss of pregabalin's inhibitory effects

**Figure 8:**
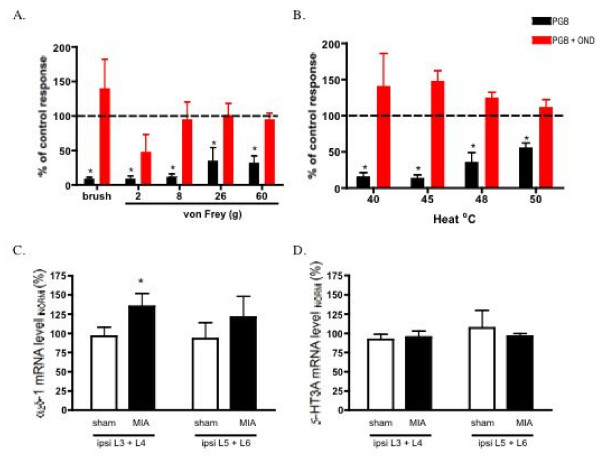
**A and B. Comparison of the effects of pregabalin alone or in the presence of ondansetron on the evoked neuronal responses to mechanical punctate (A) and thermal stimulation (B)of the peripheral receptive field in MIA rats (n = 7)**. Pregablin alone significantly inhibits the natural evoked neuronal responses. This inhibitory effect of the drug is lost when spinal 5-HT3 receptors are blocked. Data are expressed as the mean percentage of pre-drug control values ± S.E.M. * p < 0.05 significant difference compared with pre-drug baseline controls. **C. Quantification of α_2_δ-1 and 5-HT3A subunit mRNA levels in ipsilateral relative to contralateral DRGs following MIA or saline injection**. (C). Mean data for Q-PCR of α_2_δ-1 in pooled ipsilateral L3/L4 DRGs (ipsi 3+4) or pooled ipsilateral L5/L6DRGs (ipsi 5+6) from either sham (n = 10 and n = 9 respectively) or MIA animals (n = 15 or n = 7 respectively) 14 days after injection. (D). Mean data for Q-PCR of 5-HT3A in pooled ipsilateral L3/L4 DRGs (ipsi 3+4) or pooled ipsilateral L5/L6DRGs (ipsi 5+6) from either sham (n = 7 and n = 7 respectively) or MIA animals (n = 11 or n = 7 respectively). Data are normalized to the respective contralateral side and expressed as the mean percentage of control values ± S.E.M. * p < 0.05 significant difference compared with sham controls.

To test whether the active participation of spinal 5-HT3 receptors is a requirement for the efficacy of pregabalin in MIA rats, we assessed the effects of the top dose of pregabalin (10 mg/kg) in a separate group of MIA rats that had been pre-treated with spinal ondansetron. This dose of pregabalin alone produced significant neuronal inhibitions in the MIA group, but in the presence of ondansetron no longer produced any significant effects, indicating a loss of pregabalin efficacy following 5-HT3 receptor blockade (Fig. 8A, B).

### Up-regulation of α_2_δ-1 subunit in L3 + L4 DRG of MIA treated rats

Up-regulation of α_2_δ-1 subunit levels has been suggested to be correlated with the onset of allodynia and with the analgesic efficacy of gabapentinoids in a variety of models of chronic pain, such as spinal nerve ligation, HIV and chemotherapy induced neuropathies [33-39], but not in a model of radicular pain, where gabapentin still produced antinociception [40]. Therefore we measured the levels of mRNA for the α_2_δ-1 subunit in the L3 – L6 DRGs to assess whether or not there was a comparable relationship in the OA model. A small but significant increase was seen in the mRNA level for the α_2_δ-1 subunit in the ipsilateral L3 + 4 DRG (135 ± 16%) when compared with sham controls (p < 0.05, un-paired t-test). An increase in α_2_δ-1 mRNA expression was also seen for the ipsilateral L5 + 6 DRG (121 ± 28%) when compared with the appropriate contralateral DRGs although this change did not reach significance (Fig. 8C).

### 5-HT3A receptor subunit mRNA levels in lumbar DRG in MIA rats

Expression levels of the 5HT3A subunit mRNA were unchanged in the ipsilateral L3–L6 DRG compared with the contralateral side (Fig. 8D), indicating that the altered effects of 5HT3 receptor blockade are likely to be due to greater physiological activity in the descending pathways in the MIA animals and not receptor changes.

## Discussion

OA is a disease with diverse originating factors; these include a progressive degeneration of the articular cartilage, subchondral bone and weakness of joint muscles in concert with inflammatory episodes within the joint. In OA patients the pain experience is largely use-dependent and relates to the area surrounding the affected joint, however areas of referred pain also exist, for instance muscle hyperalgesia and expansion of peripheral receptive field area for pain has been demonstrated in OA patients, indicative of central sensitisation [28]. Thus pain in OA bears similarities with other chronic pain conditions, in that a variety of abnormal cellular mechanisms, both peripheral and central, underpin the pathology.

Intra-articular injection of MIA into the knee joint features pathophysiological and behavioural indices aligned with the human pain experience of OA [20-26].

Our findings corroborate earlier reports, with MIA rats displaying behavioural hypersensitive responses seen as high scores for ambulatory evoked pain and increased hind paw withdrawals to stimulation of the hind paw with von Frey filaments/acetone that had no effect on the contralateral side or in sham controls. This behavioural hypersensitivity to stimulation of the referred receptive field area is indicative of secondary hyperalgesia, which has also been reported in OA patients [23,27,28]. Furthermore, an overall increase in the baseline neuronal responses, in terms of the total number of action potentials evoked during the mechanical and thermal stimulation application period and the after-discharge response (total number of action potentials occurring after the 10-second mechanical or thermal stimulation period) is evident in the MIA group, providing evidence for central sensitization in these animals, which in turn may underlie the observed behavioural hypersensitivity. This type of hyper-responsiveness of neuronal activity has been demonstrated in a mouse model of MIA-induced OA pain [41] and in another rat model of monoarthritis [42,43].

Many studies have demonstrated a critical contribution of descending facilitation, arising from brainstem areas such as the rostral ventromedial medulla (RVM), in the development and maintenance of central sensitisation in other models of persistent pain, since pharmacological or anatomical disruption of this pathway attenuates the behavioural hypersensitivity associated with inflammatory, nerve injury and visceral models of pain, and reduces the excitability of wide dynamic range dorsal horn neurones in both acute and chronic pain states [8-18,29,44,45]. Descending pain facilitation arising from the RVM requires the activation of pronociceptive ON cells [15,46], and sensitization of RVM ON cells, which was correlated with behavioural hypersensitivity, has been demonstrated in neuropathic rats [47]. Furthermore, electrophysiological evidence from spinal cord recordings suggests activity in descending excitatory pathways is enhanced after peripheral nerve injury as demonstrated by a greater sensitivity of dorsal horn neurones to inhibition by spinal administration of ondansetron or intra-RVM administration of lignocaine [16,19]. We show here that topical spinal application of ondansetron produced marked inhibition of the evoked neuronal responses to dynamic brush and mechanical punctate stimulation in the MIA group, an effect not observed in the control group. These electrophysiological findings correlate with a recent behavioural study where the antinociceptive effectiveness of ondansetron (10 μg) was demonstrated [48].

There are a number of reports demonstrating a serotonergic facilitatory effect onto the spinal cord [44,49] through engagement of pronociceptive spinal 5-HT3 receptors in models of acute and chronic pain, with most studies using neuropathic models [12,15,17,29,50-52]. Pharmacological antagonism of physiological responses reveals the role of released transmitter acting on a particular receptor, which will depend on activity in related neuronal pathways. Thus the novel inhibitory effects of ondansetron on low threshold mechanical responses in the MIA treated group suggests adaptive changes in the activity in serotonergic pathways.

Functional 5-HT3Rs require the presence of the 5HT3A subunit [53,54], and as we did not observe any significant changes in 5HT3A receptor mRNA levels in the ipsilateral DRG of MIA rats it is unlikely that alterations in 5HT3R expression is responsible for the greater inhibitory effect of ondansetron in MIA-injected rats. Thus the data presented here suggests that increased functionality, likely augmented 5HT release, at pronociceptive spinal 5-HT3Rs is one molecular mechanism for central sensitisation underpinning the behavioural hypersensitivity seen in this model of OA pain.

The spinal source of 5-HT arises largely from the RVM (see [55]), and it is well documented that descending serotonergic influences from this brainstem area elicits bi-directional effects on spinal neurones. The facilitatory and inhibitory effects from the RVM are mediated through recruitment of RVM ON or OFF cells respectively, and there is evidence suggesting that some descending RVM ON cells use 5-HT as a neurotransmitter [12,56,57]. In addition, sensitization of pronociceptive RVM ON cells has been demonstrated in a model of peripheral nerve injury [47]. However it should be noted that pain modulation from the RVM can also be non-serotonergic, indeed immunohistological evidence suggests that pronociceptive ON cells do not contain 5-HT [58]. Another possibility then is that non-serotonergic RVM neurones may contact 5-HT containing interneurones. Nonetheless, it is possible that neuroplastic changes within the RVM may result in enhanced/altered excitatory drive mediated by 5-HT (either from direct pro-nociceptive RVM projections or via contact with 5-HT interneurones) acting on spinal 5-HT3 receptors, resulting in hyperexcitability of spinal dorsal horn neurones. Further experiments are needed to assess whether changes similar to those observed in neuropathic rats also occur in the MIA model of OA pain.

Thus our findings with spinal ondansetron in the MIA model of OA pain support previous reports and highlight the crucial link between descending serotonergic facilitation and chronic pain. Interestingly, in the MIA model the induction of 5HT3 receptor mediated facilitatory events exerted on low-threshold mechanical responses was observed suggestive of a facilitatory drive contributing to secondary hyperalgesia, which is characterized by static mechanical responses.

Systemic administration of pregabalin resulted in inhibition of the noxious evoked electrical evoked responses and also the innocuous and noxious natural evoked responses in the MIA treated rats only. A recent behavioural study by our group using the alpha2delta ligand gabapentin showed modalities of hyperalgesia being altered by gabapentin in the MIA induced model of OA pain [23]. Thus, these findings add to the growing body of evidence demonstrating the state-dependent effects of ligands at the α_2_δ-1 subunit of voltage gated calcium channels (VGCCs), actions which include unique alterations in the trafficking of the α_2_δ-1 subunit [39].

The mechanism by which gabapentinoids are able to target a ubiquitous subunit present in all VGCCs in pathophysiological states to provide analgesia yet be without antinociceptive effect under normal physiological conditions remains unclear. Alterations in VGCC function are likely to play a role, for instance N-type calcium channels acquire greater functional roles after nerve injury [59,60] and evidence exists for an upregulation of the α_2_δ-1 subunit and the N-type pore-forming α_1*B *_subunit in this pain state, which is proposed to correlate with the development of tactile allodynia and relate to the injury-specific action of gabapentin [33,35-39,61,62]. In line with this hypothesis, transgenic mice that constitutively but globally over-express the α_2_δ-1 subunit in neuronal tissues resulted in enhanced currents, altered kinetics and voltage-dependence of VGCC activation in sensory neurons; exaggerated and prolonged dorsal horn neuronal responses to peripheral mechanical and thermal stimulations; and pain behaviours [63,64].

In the present study, we observed an increase in α_2_δ-1 subunit mRNA expression in the DRG receiving afferents from the knee of the MIA group, which was comparable to findings in models of chemotherapy and varicella zoster virus induced neuropathies (approx. 46% and 38% increase respectively), and gabapentin alleviated the behavioural measures of hypersensitivity in these neuropathic animals. [34,36]. After peripheral nerve injury the upregulation is much greater in magnitude [38,39] and restricted to the denervated DRG segment which suggests that the effects of gabapentin and pregabalin on evoked responses may involve additional factors.

Indeed, upregulation of the α_2_δ subunit, is not the only determinant of the selective antinociceptive effects of gabapentinoids as the converse has also been shown, i.e. reversal of allodynic behaviour with gabapentin occurs in the absence of α_2_δ-1 subunit upregulation [40] and behavioural hyperalgesia can be observed as early as 1 day after peripheral nerve injury [65], whilst α_2_δ-1 upregulation is only evident after 7 days [37]. Another mechanism proposed to be permissive for the antinociceptive effectiveness of α_2_δ-1 ligands and their ability to differentiate pathological states is the increased active participation of descending serotonergic facilitation of spinal neuronal activity via activation of spinal 5-HT3 receptors [19,29]. Interruption of descending facilitation resulted in a loss of antinociceptive effectiveness of gabapentin/pregabalin in neuropathic rats [17,29], whilst activating spinal 5-HT3 receptors in naïve control animals enabled gabapentin to produce marked inhibitions of evoked neuronal activity where previously, in the absence of 5-HT3 activation, the drug had little or no effect [29]. In healthy humans, capsaicin induced hyperalgesia is sensitive to gabapentin in terms of both psychophysics and fMRI activation in brainstem and other brain areas, conditions where no time for upregulation of the α_2_δ-1 subunit could occur [30,66]. The findings of the present study parallel those seen in the spinal nerve ligation model and lend support for the concept that serotonergic circuits play a role in regulating the state dependent antinociceptive effects of gabapentinoids, since we observed an increased sensitivity of spinal neurons to the inhibitory actions of spinal 5-HT3 antagonism, indicating enhanced descending facilitation in the OA group, and critically, the state dependent inhibition of neuronal responses with pregabalin were lost when 5-HT3 facilitation was blocked.

## Conclusion

Overall, the data presented here demonstrate an altered physiology of deep dorsal horn wide dynamic range neurons in the MIA group, indicating a link between increased neuronal activity and behavioural hypersensitive responses in these animals. The novel and increased sensitivity of mechanical and thermal evoked neuronal responses to the inhibitory effects of spinal ondansetron suggests an enhanced functionality at pronociceptive spinal 5-HT3 receptors. Thus our findings would implicate these changes as contributory molecular substrates underlying central sensitisation, a feature of many chronic pain states, in osteoarthritis. Finally, spinal ondansetron prevented the antinociceptive actions of pregabalin, suggesting that descending facilitation, alongside the upregulation in the expression of α_2_δ-1 subunit of VGCCs, is an important mechanism which enables the inhibitory actions of pregabalin in this model of OA pain.

OA represents one of the largest clinical costs to healthcare in the western world [67]. The associated pain is the main symptom of complaint in patients with a pan-European survey showing that nearly a third of patients suffering from chronic pain presented with OA as the primary cause [68]. Therefore unraveling the cellular and molecular mechanisms underlying the pain state is of major clinical importance in the development of more clinically effective drugs.

## Methods

### Induction of Osteoarthritis

Osteoarthritis was induced by injecting 2 mg monosodium iodoacetate in 25 μl of 0.9% saline through the infrapatellar ligament of the knee in anesthetised Sprague Dawley rats (130–150 g). Sham animals were injected with sterile 0.9% saline only. Following injection animals were allowed to recover and then re-housed in cages under a 12-h alternating light/dark cycle with ad libitum access to food and water.

### Behavioural assessment

Behavioural responses to mechanical and cooling stimulation of the ipsi- and contralateral hind paws were recorded over a two-week period, as was performance on a rotarod. Briefly, animals were left to acclimatize to the testing area for 30 min before testing, sensitivity to mechanical puncatate stimulation was then assessed through measurement of the number of foot withdrawal responses to a trial of 10 applications of calibrated von Frey filaments with increasing bending force of 1 and 6 g to the plantar surface of each hind paw. Cold sensitivity was similarly assessed as the number of withdrawals out of a trial of 5 applications of a drop of acetone to the plantar surface of ipsilataeral and contralateral hind paws. Withdrawal frequency was quantified as = (number of foot withdrawals/10 or 5 trials as appropriate).

Behavioral signs of ambulatory-evoked pain were assessed preoperatively and on postoperative days -3, 2, 4, 7, 9 11 and day 14 using the Ugo Basile model 7750 Rotarod (Linton Instruments, Diss, Norfolk, UK). The apparatus was set to accelerate from 0–20 revolutions per minute (rpm) over 60 s and the time maintained on the beam before falling was recorded (with a maximum cut-off of 180 s). The general ambulation of the animal was also observed and scored as follows: 0 = no limp, 1 = slight limp, 2 = marked limp and decreased use of ipsilateral limb and 3 = avoidance of use of ipsilateral limb. Previous studies from our laboratory have validated the use of the rotarod test as a reliable means for assessing ambulatory evoked pain [23,69-71]. Only rats scoring between 60 and 120 s in training sessions before surgery were used for experimentation.

### Electrophysiology

Two weeks after MIA injection *in vivo *electrophysiological studies were performed (post-operative days 15 – 19) as previously described. Briefly, animals were anesthetised and maintained for the duration of the experiment with isofluroane (1.5–1.7%) delivered in a gaseous mix of N_2_O (66%) and O_2 _(33%). A laminectomy was performed to expose the L4–5 segments of the spinal cord. Extracellular recordings were made from ipsilateral deep dorsal horn neurones (lamina V-VI) using parylene coated tungsten electrodes (A-M Systems, USA). All the neurones recorded in this study were WDR since they all responded to both light touch and noxious inputs (pinch and noxious heat); further all neurones responded to natural stimuli in a graded manner with coding of increasing intensity.

The evoked response to a train of 16 transcutaneous electrical stimuli (2 ms wide pulses, 0.5 Hz) applied at 3 times the threshold current for C-fibre activation of the dorsal horn cell. The train of electrical stimuli was delivered via stimulating needles inserted into the peripheral receptive field, following which a post-stimulus histogram was constructed. Responses evoked by Aβ – (0–20 ms), Aδ – (20–90 ms) and C-fibres (90–350 ms) were separated and quantified on the basis of latency. Responses occurring after the C-fibre latency band were taken to be the post-discharge of the cell (350–800 ms).

The centre of the peripheral receptive field was also stimulated using mechanical punctate and thermal stimuli (von Frey filaments, 2, 8, 26 and 60 g and heat, applied with a constant water jet, 35, 40, 45, 48 and 50°C) Application of each von Frey hair was separated by a minimum interval period of 5 10 seconds, and longer for very responsive neurons at the higher intensity range. Application of each subsequent heat stimulus was separated by a minimum period of 1 minute. All natural stimuli were applied for a period of 10 seconds per stimulus. Data was captured and analysed by a CED 1401 interface coupled to a Pentium computer with Spike 2 software (Cambridge Electronic Design; PSTH and rate functions).

On average, between 1 to 3 neurones were recorded from each animal in order for their baseline responses to peripheral stimuli (detailed above) to be characterized. Pharmacological assessment was carried out on the final neuron recorded in each animal, i.e. a drug study was carried out on one neuron only per animal. The testing procedure was carried out every twenty minutes and consisted of a train of electrical stimuli followed by natural stimuli as described above. Following three consecutive stable control trials (< 10% variation for the C-fibre evoked response, and < 20% variation for all other parameters) neuronal responses were averaged to give the pre-drug control values.

### Drug administration

Ondansetron (Zofran TM Glaxo-Wellcome) was diluted with 0.9% saline solution to give doses of 10, 50 and 100 μg/50 μl, and was administered via topical spinal application with a Hamilton syringe. Pregabalin (a gift from Pfizer, Sandwich, UK) was dissolved in 0.9% saline solution to give doses of 0.3, 3 and 10 mg/kg, which were administered via subcutaneous injection in the scruff of the back of the neck.

The effect of each dose (ondansetron or pregabalin) was followed for over an hour, with tests carried out at 20, 40 and 60 minutes before subsequent drug applications were made and effects again followed for an hour.

### Quantitative PCR – α_2_δ-1 and 5-HT3 receptor subunit mRNA levels

Pain associated with peripheral nerve injury and also the analgesic efficacy of gabapentinoids has been correlated with a marked upregulation of the α_2_δ-1 subunit of voltage gated calcium channels in rats [33,35,37-39]. Furthermore there is evidence for neuropathic pain associated with osteoarthritis [72]. Therefore we analysed the α_2_δ-1 subunit mRNA levels in DRG for the primary afferents innervating the knee joint and hindpaw (L3–L6) in MIA rats. Additionally, the 5-HT3A subunit mRNA was similarly quantified. We verified that the knee afferents arise from L3 (5.2% of total cells) and L4 (2.9%) as compared to L5 (0.5%) by counting labelled cells after intra-articular injection of Fast Blue (10 μl 1%).

Following the last behavioural testing day (post injection day 14), MIA-treated animals were decapitated and L3–6 DRGs ipsilateral and contralateral to injection were harvested and kept at -80°C. The methods for quantitative PCR (Q-PCR) were essentially as described previously (Donato et al., 2006). Briefly, RNA was extracted from ipsi- or contralateral L3 – L6 pulverized frozen DRGs, 14 d after injection of MIA or saline into the knee joint. RNA was isolated using RNeasy columns (Qiagen), including an on-column DNase step. Reverse transcription was carried out on 1 μg RNA using the iScript kit with random primers (BioRad, Hercules, CA). Q-PCR was performed with an iCycler (BioRad) using the iQ SYBR supermix (Biorad). For each set of primers and for every experiment a standard curve was generated using a serial dilution of reverse-transcribed RNA from the combined samples. The following Q-PCR primers were used: rat GAPDH (AF106860) 5'-ATGACTCTACCCACGGCAAG-3' (forward), 5'-CATACTCTGCACCAGCATCTC-3' (reverse); rat α_2_δ-1 (NM012919) 5'-AGCCTATGTGCCATCAATTAC-3', 5'-AGTCATCCTCTTCCATTTCAAC-3'; rat 5HT3A (NM024394) 5'-AGCCTTGACATCTATAACTTCC-3', 5'-TCCGACCTCACTTCTTCTG-3'.

## Data analysis

All data are presented as mean ± standard error of mean (S.E.M.). Behavioural data were analysed using the non-parametric Mann-Whitney *U*-test. Analysis of electrophysiological was as follows: Differences between groups with respect to pre-drug baseline responses were analysed using unpaired Student's t-test to compare the cell depth and electrically evoked responses; a two-way ANOVA followed by Bonferroni post-hoc test was used to compare the baseline mechanical and thermal evoked neuronal responses. Drug effects were expressed as the mean maximal evoked neuronal response for each dose. A one-way analysis of variance with repeated measures (RM-ANOVA) was used to evaluate drug effects (ondansetron or pregabalin) on the evoked responses to each stimulus (electrical, mechanical and heat) in MIA or vehicle treated rats. Where a significant effect was seen with increasing dose, Dunnett's post hoc tests where then used to assess individual dose effects compared with pre-drug baseline controls. An unpaired t-test on normalised data was used to compare drug effects between experimental groups. Analysis of Q-PCR data was as follows: For each set of primers and for every experiment a standard curve was generated using a serial dilution of reverse-transcribed RNA from the combined samples. Data were normalized for expression of glyceraldehyde-3-phosphate dehydrogenase (GAPDH) mRNA. Data from the ipsilateral DRG were then normalized to their respective contralateral DRG and given as the mean ± SEM. The cell bodies of the primary afferents supplying the knee joint predominate in L3 & L4 DRG and the L5 & 6 DRG are representative predominantly of afferent cell bodies of the referred receptive field area, therefore the q-PCR data was pooled into two groups (L3+L4 and L5+L6). Statistical significance between OA and sham animals was determined by a Student's un-paired t-test. All statistical testing was performed by using Prism 4.0 software (Graphpad/Prism, San Diego, Calif). Level of significance was set at * P < 0.05.

## Competing interests

The authors declare that they have no competing interests.

## Authors' contributions

WR and AHD conceived, designed and performed the experiments, analysed and wrote the manuscript. CSB performed and analysed some of the experiments. KB and JLV performed some of the experiments. All authors approved the final manuscript.
